# Different Metabolic Pathways Are Involved in Response of *Saccharomyces cerevisiae* to L-A and M Viruses

**DOI:** 10.3390/toxins9080233

**Published:** 2017-07-25

**Authors:** Juliana Lukša, Bazilė Ravoitytė, Aleksandras Konovalovas, Lina Aitmanaitė, Anzhelika Butenko, Vyacheslav Yurchenko, Saulius Serva, Elena Servienė

**Affiliations:** 1 Laboratory of Genetics, Institute of Botany, Nature Research Centre, Akademijos str. 2, Vilnius LT-08412, Lithuania; juluksa@gmail.com (J.L.); bazilerav@gmail.com (B.R.); 2 Department of Biochemistry and Molecular Biology, Institute of Biosciences, Vilnius University, Saulėtekio al. 7, Vilnius LT-10257, Lithuania; aleksandras.konovalovas@gf.vu.lt (A.K.); lina.aitmanaite@gf.vu.lt (L.A.); saulius.serva@gf.vu.lt (S.S.); 3 Life Science Research Centre and Institute of Environmental Technologies, Faculty of Science, University of Ostrava, Chittussiho 10, 710 00 Ostrava, Czech Republic; rolando24@ya.ru (A.B.); vyacheslav.yurchenko@osu.cz (V.Y.)

**Keywords:** *Saccharomyces cerevisiae*, dsRNA viruses, host gene expression, RNA-Seq

## Abstract

Competitive and naturally occurring yeast killer phenotype is governed by coinfection with dsRNA viruses. Long-term relationship between the host cell and viruses appear to be beneficial and co-adaptive; however, the impact of viral dsRNA on the host gene expression has barely been investigated. Here, we determined the transcriptomic profiles of the host *Saccharomyces cerevisiae* upon the loss of the M-2 dsRNA alone and the M-2 along with the L-A-lus dsRNAs. We provide a comprehensive study based on the high-throughput RNA-Seq data, Gene Ontology and the analysis of the interaction networks. We identified 486 genes differentially expressed after curing yeast cells of the M-2 dsRNA and 715 genes affected by the elimination of both M-2 and L-A-lus dsRNAs. We report that most of the transcriptional responses induced by viral dsRNAs are moderate. Differently expressed genes are related to ribosome biogenesis, mitochondrial functions, stress response, biosynthesis of lipids and amino acids. Our study also provided insight into the virus–host and virus–virus interplays.

## 1. Introduction

Mycoviruses are common in fungi and typically possess dsRNA genome [1]. Fungal viruses lack the extracellular phase; they are inherited vertically either after cell division or through mating with a donor cell [2,3]. It has been demonstrated that genes of dsRNA viruses (i.e., *Totiviridae* and *Partitiviridae*) have widespread homologs in the nuclear genomes of eukaryotic organisms, such as plants, arthropods, fungi, nematodes, and protozoa, suggesting that viral genes might have been transferred horizontally from viral to eukaryotic genomes [3,4]. *Totiviridae* viruses in yeast are generally associated with symptomless and persistent infections [1], affecting host fitness in various ways by changing the virulence of fungal plant pathogens [5,6] and/or inducing toxin production [7,8,9].

Budding yeast is one of the best-described unicellular eukaryotic model organisms. It hosts *Totiviridae* dsRNA viruses such as L (L-A-1, L-A-2, L-A-28, L-A-lus, and L-BC) and M (M-1, M-2, M-28, and M-lus) [2,10,11]. These relatively small (around 4.6 kb for L; 1.5–2.3 kb for M) dsRNA determine yeast killer phenotype discovered almost 50 years ago [12]. The genome of the L-A virus typically encompasses two ORFs, one for the capsid protein another one for the RNA-dependent RNA polymerase, latter encoded as a frameshift-dependent read-through protein. M dsRNA genome contains a single ORF encoding precursor of the toxin, processed into the secreted toxin and the preprotoxin providing self-immunity [2]. The killer phenotype, which may also be determined by chromosome- or plasmid-encoded toxins, is known to be widespread in nature [13]. The killer system confers increase competitiveness toward sensitive strains contending for resources in the environment [14]. This attractive property encouraged the application of killer yeast in many industrial and therapeutic processes [15,16,17,18,19,20]. Screening for yeast genes related to altered cell susceptibility to certain M dsRNA-encoded killer toxins (K1, K2, and K28) addressed the killing mechanisms in further details [21,22,23]. In contrast, the role of the L virus, maintained in yeast cells lacking killer phenotype, remains obscure. There is no current agreement whether the residual L virus is a relic of a killer system, which has lost the M dsRNA, or if it grants yet uncovered benefits for the host cell. One example of such beneficial relationship is dsRNA of mycoviruses, providing the host with virulence-associated traits such as increase in growth rate and sporulation [5]. Loss of the M dsRNA would be unfavorable in killer yeast populations, since a virus-free cell would be vulnerable to the toxin produced by the surrounding cells [14].

Yeasts and their dsRNA viruses provide a convenient model system for studying the host–virus interactions. In terms of phylogeny, recent studies revealed killer strains to be more related to each other than to the non-killer kins [24], suggesting co-adaptation of natural yeast killer strains and their viruses [25]. Gene expression analysis has been employed to examine the impact of viral infections on host cells of various organisms (bacteria [26,27]; plants [28]; and animals [29,30] including human [31,32]). Previously, transcriptional responses related to fungal viruses have been investigated [33,34,35]. Meanwhile, data on transcriptional responses induced by the elimination of dsRNA viruses from their yeasts hosts are scarce. To the best of our knowledge, the only report concerned the impact of yeast L-A-1 and M-1 dsRNA viruses on the host transcription alteration of *S. cerevisiae* utilized a microarray approach to assay transcriptional changes [36]. 

In this study, we provide an overview of yeast (*S. cerevisiae*) gene expression changes in M437 strain cured of the M-2 exclusively or from both M-2 and L-A-lus dsRNA viruses analyzed by Next-Generation Sequencing technique. Differently expressed genes were subjected to Gene Ontology (GO) analysis and their physical and/or functional interaction networks were established. Our results provide new insights into the virus–host and virus–virus interactions and the bases of co-adaptation.

## 2. Results

### 2.1. Differential Gene Expression Induced by Elimination of Viral DsRNA(s)

To study the transcriptional response to the loss of viruses, yeast cells lacking either M-2 (M437 [L+M−]) or both L-A-lus and M-2 dsRNA viruses (M437 [L−M−]) were generated. The absence of corresponding dsRNA(s) in particular cells was confirmed by functional tests, dsRNA gel electrophoresis and RT-PCR (Figure S1). 

To elucidate changes in gene transcription of the yeast strain induced by different sets of dsRNA viruses, whole transcriptome profiling was performed. Transcriptional changes induced by the absence of viral dsRNA(s) in M437 [L+M−] and M437 [L−M−] cells were compared to the wild type *S. cerevisiae* M437 [L+M+] K2-killer strain, which naturally possesses L-A-lus and M-2 dsRNAs. Hereafter, upregulation indicates higher and downregulation lower transcription levels in dsRNA(s)-free cells compared to M437 [L+M+].

In total, 486 genes were shown to be differentially expressed (at least 1.5-fold change; Benjamini and Hochberg False Discovery Rate corrected, with *p* < 0.05 significance level) after eliminating the M-2 dsRNA (Table S1). Out of these, 239 and 247 genes were significantly up- and downregulated compared to the reference strain M437 [L+M+], respectively. Elimination of both M-2 and L-A-lus dsRNAs affected transcriptional changes of 715 genes (about 1.5 times more genes compared to the M-2 loss alone) (Table S1). In M437 [L−M−] cells, 291 and 424 genes were up- and downregulated, respectively. Interestingly, while elimination of M-2 dsRNA caused upregulation and downregulation of similar number of genes, simultaneous removal of both viruses increased the proportion of negatively regulated genes about 1.5 times, compared to the M-2-free cells.

**Figure 1:**
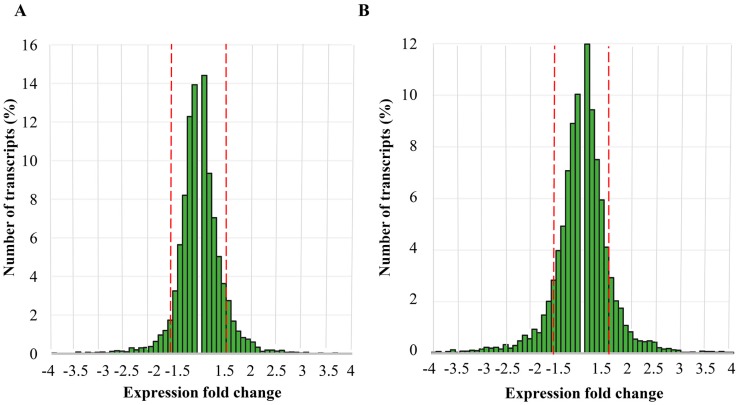
Distribution of expression fold differences induced by the loss of viral dsRNA(s). Distribution of expression fold changes induced by elimination: of M-2 dsRNA (L+M−) (**A**); or of both L-A-lus and M-2 dsRNAs (L−M−) (**B**). Range of represented fold changes is from −4 to 4. Red dashed lines mark the threshold of significant fold change value (1.5-fold change) in our analyses. Bar heights represent percentage of genes showing corresponding fold change compared to the *S. cerevisiae* S288C reference genome (6008 genes in total).

Most changes in host gene expression in response to the loss of viral dsRNA(s) were moderate and did not exceed the limit of four-fold change (Figure 1). 

Only 19 genes in M437 [L+M−] and 22 genes in M437 [L−M−] cells were upregulated four-fold or more, while downregulation for more than four-fold was documented for 39 genes in M437 [L+M−] and 66 genes in M437 [L−M−] cells in comparison to the reference strain. 

Representative examples of the up- and down-regulated genes were evaluated by qRT-PCR confirming the data obtained in whole-transcriptome profiling experiments (Figure S2). 

### 2.2. Transcriptional Response to M-2 DsRNA Elimination

To characterize the cellular activities connected with the viral dsRNA elimination, we calculated the enrichment of “biological process”, “cellular component” and “function” gene ontology (GO) terms associated with genes of altered transcription. We identified 363 and 231 statistically enriched GO terms of genes altered by the elimination of M-2 and both M-2 and L-A-lus, respectively (Tables S2–S4). 

**Figure 2:**
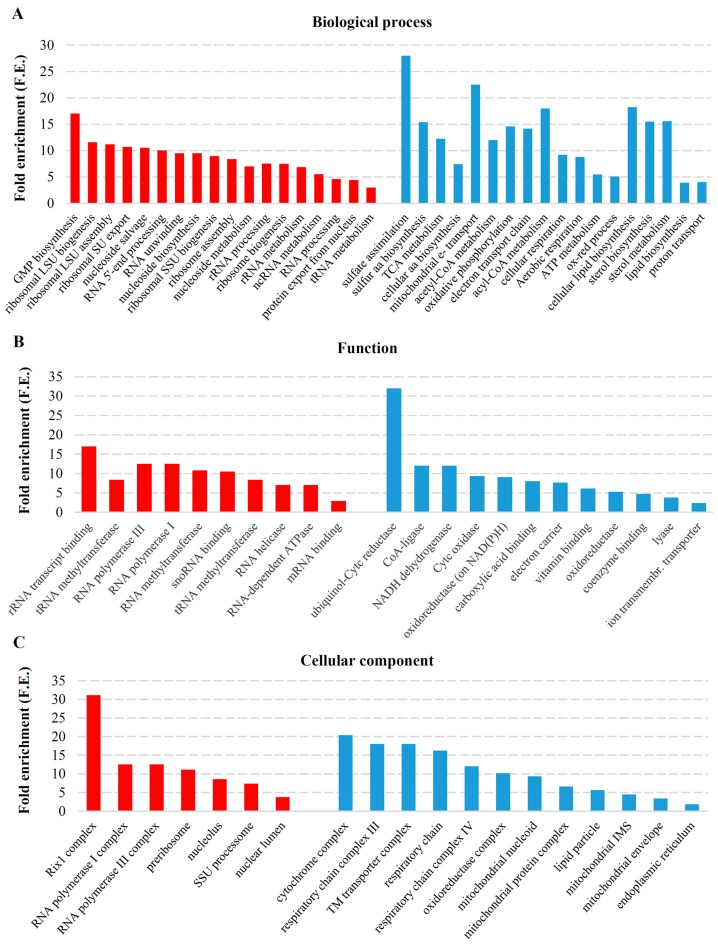
Statistically significant enriched gene ontology terms associated with the functioning of M-2 virus. Fold enrichment (F.E.) was calculated by dividing the frequency of specific gene cluster to the total frequency for each GO term, according to the data presented in Tables S2–S4: (**A**) enriched GO terms associated with biological processes; (**B**) functions; and (**C**) cellular components. Color coding is as follows: red, upregulated genes; blue, downregulated. snoRNA: small nucleolar RNA; rRNA: ribosomal RNA; SSU: small ribosome subunit; LSU: large ribosome subunit; TM: transmembrane; IMS: intermembrane space; Cytc-cytochrome c.

In transcriptional response to the M-2 dsRNA elimination, RNA-related processes such as ribosome biogenesis and assembly (Fold enrichment (F.E.) of 7.5 and 8.4, respectively); metabolic processes of rRNA, ncRNA, and tRNA (F.E. of 6.9, 5.5, and 3, respectively); and RNA 5′-end and rRNA processing (F.E. of 10.0 and 7.5, respectively) were positively stimulated (Figure 2A). 

Nucleoside biosynthetic and metabolic processes were highly enriched (F.E. of 9.5 and 7, respectively), including GMP biosynthesis and nucleoside salvage (F.E. of 17 and 10.5, respectively). Genes involved in RNA secondary structure unwinding, ribosomal subunit and protein export from the nucleus (F.E. of 9.5, 10.7 and 4.4, respectively) were upregulated in response to the M-2 virus loss. Functional allocation of the upregulated genes fell into the activities of RNA methyltransferase (F.E. of 10.8), RNA-dependent ATPase and RNA helicase (F.E of 7 of each), and RNA polymerase I and III (F.E of 12.5 of each) (Figure 2B, Table S3). GO terms defining localization were consistent with the assigned RNA-related processes (Table S4). Enriched cellular component terms included nuclear lumen (F.E. of 3.8), nucleolus (F.E. of 8.53) and preribosome (F.E. of 11.15). Complexes such as DNA-directed RNA polymerase I and RNA polymerase III (F.E. of 12.5 of each) and Rix1 (F.E. of 31.05) were also highly represented (Figure 2C, Table S4). 

Elimination of the M-2 dsRNA resulted in downregulation of genes involved in distinct biological processes (Figure 2A, Table S2). Genes involved in energy production (electron transport chain and cellular respiration with respective F.E. of 14.2 and 9.2), cellular lipid biosynthesis and sterol metabolism (F.E. of 18.3 and 15.6, respectively), and biosynthesis of cellular amino acids (F.E. of 7.4) were downregulated. The decreased activity of mentioned above systems are consistent with diminished demand for energy, amino acids and lipids in the absence of the M-2 virus. Sulfate assimilation (F.E. of 28) and sulfur amino acid biosynthetic processes (F.E. of 15.4) along with mitochondrial electron transport (ubiquinol to cytochrome c) (F.E. of 22.5), acyl-CoA, acetyl-CoA and sterol metabolic processes (F.E. of 18.01, 12.0 and 15.6, respectively) were among the most enriched GO terms (Figure 2A). Function ontology confirmed downregulation of electron carrier, oxidoreductase, cytochrome c oxidase, NADH dehydrogenase and CoA-ligase with respective F.E. of 7.6, 5.3, 9.3, 12 and 12 activities, related to mitochondria (Figure 2B, Table S3). Localization enrichment also supports the importance of mitochondria in the functioning of M-2 virus (mitochondrial respiratory chain (F.E. of 17.3), cytochrome complex (F.E. of 20.3), mitochondrial envelope (F.E. of 3.4), mitochondrial protein complex (F.E. of 6.6), and respiratory chain complexes III and IV (F.E. of 18 and 12, respectively) were all downregulated) (Figure 2C, Table S4). 

### 2.3. Transcriptional Response to Elimination of Both M-2 and L-A-lus

**Figure 3:**
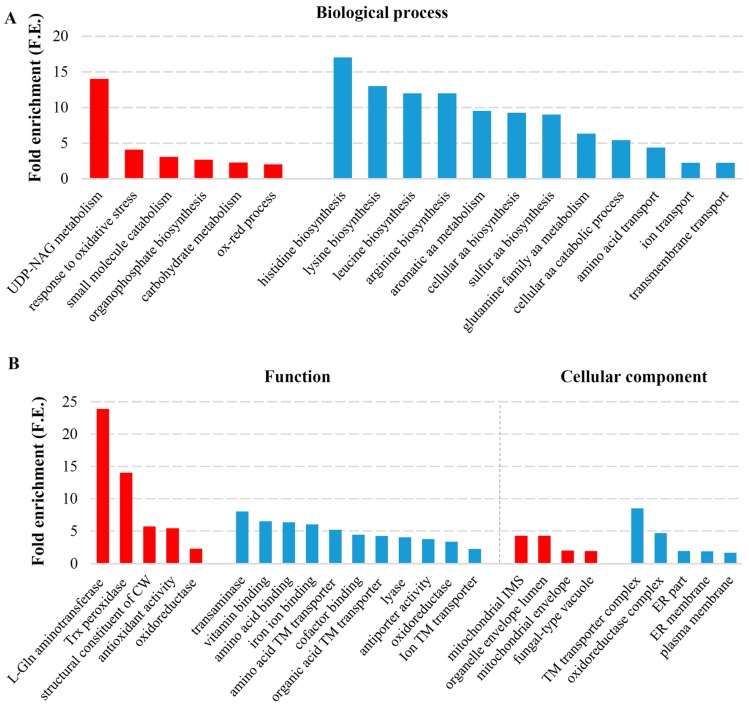
Statistically significant enriched gene ontology terms associated with the functioning of both M-2 and L-A-lus viruses: (**A**) statistically significant enriched GO terms associated with biological process; and (**B**) functioning and cellular components. Red color represents upregulated genes in both virus-free cells, blue-downregulated genes. Fold enrichment (F.E.) was calculated by dividing the frequency of specific gene cluster to the total frequency for each GO term, according to the data presented in Tables S2–S4. Trx: thioredoxin; CW: cell wall; IMS: intermembrane space; TM: transmembrane; UDP-NAG: UDP-N-acetylglucosamine; L-Gln: L-glutamine.

While genes upregulated in the M-2-free cells positioned within highly enriched and quite specific categories, elimination of the L-A-lus and M-2 dsRNAs triggered fewer number of broader processes in terms of upregulation (Figure 3A). More than 50 out of 291 upregulated genes in M437 [L−M−] cells are listed as uncharacterized. This is the largest number of uncharacterized genes among all four sets of differently expressed genes described in our study. Enriched GO terms of positively regulated genes are related to stress response, namely cellular response to oxidative stress, oxidation-reduction process, carbohydrate metabolic process and UDP-N-acetylglucosamine metabolic process (F.E. of 4.06, 2.02, 2.27 and 14, respectively) (Figure 3A). Proteins involved in UDP-N-acetylglucosamine metabolic process are related to cell wall biogenesis (synthesis of chitin, GPI anchor and mannoproteins) [37]. Response to stress is supported by function ontology enrichment of structural constituent of cell wall (F.E. of 5.7) and related L-glutamine aminotransferase activity (F.E. of 23.9), as well as antioxidant (F.E. of 5.4) and related thioredoxin peroxidases (F.E. of 14) (Figure 3B). 

Downregulated genes are involved in highly similar processes in response to elimination of the M-2 dsRNA alone or both L-A-lus and M-2 viruses (Figure 2A and Figure 3A). M437 [L+M−] and M437 [L−M−] cells share negative regulation of genes related to more than 50% of total number of enriched processes (assigned to downregulated genes in both cell types), namely cellular amino acid biosynthetic process (F.E. of 7.4 and 9.3, respectively), cellular lipid biosynthetic process (F.E. of 18.25 and 13, respectively), cellular respiration (F.E. of 9.2 and 3, respectively) (Figure 2 and Figure 3). Total number of downregulated genes was higher in M437 [L−M−] than in M437 [L+M−] cells (424 and 247 genes, respectively), indicating that the fold enrichment values tend to be lesser despite the greater number of genes assigned to the same process. 

Nevertheless, judging by F.E. values and assigned number of genes, amino acid biosynthesis was clearly downregulated in M437 [L−M−] in a greater extent than in M437 [L+M−] cells. The expression of genes related to overall and specific amino acid biosynthesis (arginine (F.E. of 12), histidine (F.E. of 17), leucine (F.E. of 12), and lysine (F.E. of 13)) and transport (F.E. of 4.4) were highly repressed upon elimination of both viruses (Figure 3A). Negatively regulated processes were related to ATP synthesis (such as aerobic respiration, tricarboxylic acid metabolism, mitochondrial electron transport (ubiquinol to cytochrome c) and proton transport with respective F.E. of 8.8, 11.3, 22.5, 4.1) were more specific to M437 [L+M−] cells (Figure 2A). Functional ontology confirmed downregulation of oxidoreductases, transaminases and lyases (F.E. of 3.3, 8.0, 4.0, respectively), functions of transmembrane transporters (of amino acids, organic acids and ions, with respective F.E. 5.2, 4.2 and 2.2) and binding activities (of various cofactors (F.E. 4.4), amino acids (F.E. 6.3), vitamins (6.5)) were more typical to M437 [L−M−] cells (Figure 3B). However, more specific assignment of oxidoreductase function confirms that oxidoreductase activity in M437 [L−M−] cells is related to synthesis of amino acids and sterols by acting on CH-NH, CH-NH_2_ groups and on paired donors, with incorporation or reduction of molecular oxygen (Table S3). In M437 [L+M−] cells, the oxidoreductase activity was linked to ubiquinol and cytochrome c, by acting on CH-CH and CH-OH groups of donors (Table S3). This indicates that M-2 disposal significantly reduces cellular energy costs, but L-A-lus dsRNA possessing cells still require elevated synthesis of amino acids comparing to that of dsRNA-free cells.

### 2.4. Gene Products Involved in M-2 and L-A-lus Virus Biology Are Physically and Functionally Highly Interconnected In Vivo

**Figure 4:**
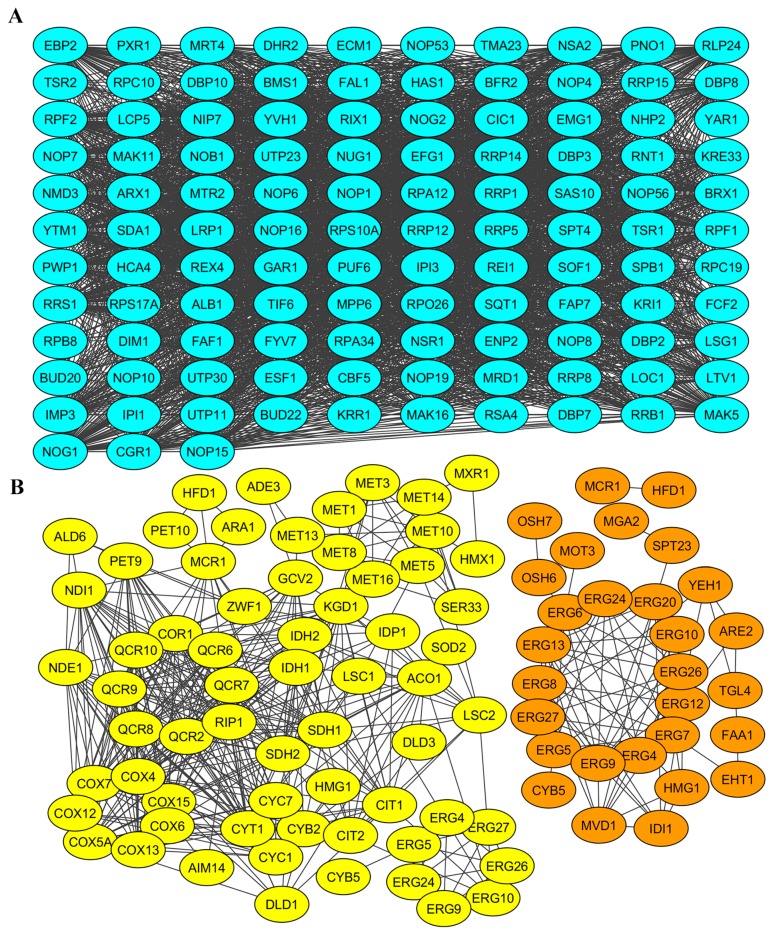
Networks of interconnected gene products involved in viral M-2 dsRNA biology. Networks of physically and/or functionally interacting gene products were established with STRING (see Materials and Methods). Gene products are depicted as color-coded nodes, according to cellular processes, and are connected by edges. Color coding is as follows: blue, ribosomal biogenesis; yellow, oxidation-reduction processes and energy generation; orange, lipid metabolism. (**A**) Gene products upregulated in response to loss of M2 virus; and (**B**) downregulated gene products in M2 virus-free cells.

**Figure 5:**
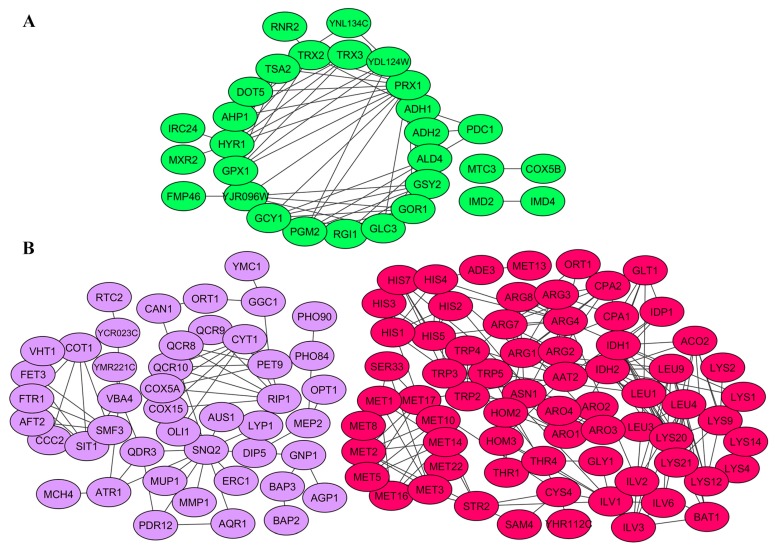
Networks of interconnected gene products involved in biology of L-A-lus and M-2 dsRNAs. Physical and/or functional networks were established with STRING (see Materials and Methods). Gene products are depicted as color-coded nodes, according to cellular processes, and are connected by edges. Color coding is as follows: green, oxidation-reduction and stress response; purple, transmembrane transport; red, amino acid biosynthesis. (**A**) Gene products upregulated in response to loss of M2 and L-A-lus viruses; and (**B**) downregulated gene products in M2 and L-A-lus-free cells.

The physical and/or functional interaction of gene products identified in RNA-Seq. analysis was addressed next. For this, a STRING database of known and predicted protein interactions was used to analyze gene products involved in processes altered by elimination of M-2 (Figure 4) and both L-A-lus and M-2 viral dsRNAs (Figure 5). 

Products of genes with altered expression level in M-2-free cells and related to ribosome biogenesis, oxidation-reduction and lipid metabolism were shown to be highly interconnected (Figure 4). Interconnections formed highly-reliable hub between members of ribosome biogenesis group. Most of the genes encoding these proteins are essential. They are related to ribosomal RNA processing (Rrp1, -5, -12, and -14); ATP-dependent RNA helicases of the DEAD-box protein family (Dbp2, -8, and -10); components of the Rix1 complex and pre-replicative complexes Ipi1 and Ipi3; components of RNA polymerases (Rpb8, and Rpc10 and -19); many nucleolar proteins (e.g., Nop1, -4, -7, -8, and -10); and others. In terms of fold change, *NSR1*, *RRS1* and *ALB1* were the most (almost three-fold) upregulated genes in this group. Among the closely interconnected gene products involved in ribosome biogenesis, we noted those that are also required for maintenance of M-1 dsRNA (Mak5, Mak11, Mak16) [38,39,40] and involved in susceptibility to K1 (Kre33) [21] or both K1 and K2 killer toxins (Fyv7) [23]. 

The products of downregulated genes in M437 [L+M−] cells showed interconnections of high confidence level between members related to cellular energetic processes (Figure 4B). Most genes are directly involved in mitochondrial structure and function, such as cytochrome b and c (*CYB2*, *CYB-5*, *CYT1*, *CYC1* and *CYC7*), cytochrome c oxidase complex subunits (*COX4*, *COX5A*, *COX6–7*, *COX12–13* and *COX15*), and ubiquinol cytochrome c reductase subunits (*COR1*, *RIP1*, *QCR2* and *QCR6–10*) encoding genes. There are also many genes involved in various cellular processes associated with the generation of energy, such as sulfate assimilation and methionine metabolism (*MET1*, *-3*, *-5*, *-8*, *-10*, *-13*, *-14* and *-16*), lipid metabolism and genes that may be related to stress response (*MRX1*, *SOD2*, *HMX1*, *NDI1*, *MIX17*, and *ZWF1*). Among downregulated genes, clustered into group associated with oxidation-reduction processes and generation of energy, a sub-network of highly interconnected 29 gene products involved in lipid metabolism was evident (high confidence level). Most of them were associated with ergosterol biosynthesis and metabolism (Mcr1; Erg4, -5, - 9, -10, -24, -26, and -27; Osh6 and -7; Idi1; and Mvd1) and homeostasis of lipid particles (Are2, Yeh1, Tgl4, Eht1, Faa1) (Figure 4B). TAR1 is the most downregulated (almost nine-fold) gene in the group of cellular energetic processes. Interestingly, it is encoded in the antisense strand of the nuclear 25S rDNA. *TAR1* and rDNA transcription might be inversely regulated consistent with the finding that ribosome biogenesis is highly upregulated in M-2 dsRNA-free cells. Even though Tar1 function is still unknown, this regulation could provide means to coordinate rDNA transcription and mitochondrial function in response to changing cellular needs or energy demands [41].

Among the gene products, differently expressed in M437 [L−M−] cells, three interconnected groups related to oxidation-reduction, amino acid biosynthesis and transmembrane transport were built (Figure 5). Products of represented genes possess high (0.7) interaction confidence level. 

Numerous upregulated genes assigned to oxidation-reduction group encoded reductases (Gor1, Mxr2, and Rnr2), dehydrogenases (Imd2, Imd4, Adh1, Adh2, Ald4, and Gcy1) and peroxidases (Tsa2, Hyr1, and Gpx1). *PRX1*-encoded mitochondrial peroxiredoxin with thioredoxin peroxidase activity has most interactions in the group and is known to be induced during oxidative stress (Figure 5A). 

Importantly, elimination of either M-2 or M-2 and L-A-lus dsRNA altered expression of several genes, related to oxidation-reduction processes. However, these genes do not overlap and were shown to be involved in distinct processes: in M-2 dsRNA-free cells they are associated with energy generation and in M-2 and L-A-lus free cells they are related to stress response. 

Interactions among gene products of downregulated genes related to amino acid biosynthesis were represented by the highest confidence level (Figure 5B). Certain enzymes, involved in biosynthesis of more than one amino acid (e.g., enzymes of TCA cycle (Aco2, Idh1, and Idh2), Ade3 (required for biosynthesis of methionine and histidine), Hom2 and -3 (for methionine and threonine biosynthesis), Aro1–4 (for aromatic amino acids), and others) strengthen interactions among group members. Connections between gene products, related to biosynthesis of glutamate (Idh1 and Glt1), arginine (Ort1, Arg1–4, Arg5–6, Arg7–8, and Cpa1–2), methionine and cysteine (Met1–3, -5, -8, -10, -13, -14, -16, -17, and -22; Sam4; Cys4; and Str2), leucine, isoleucine and valine (Leu1, -3, -4, and -9 and Ilv1–3, and -6), serine and glycine (Ser33 and Gly1), threonine (Thr1 and -4), aromatic amino acids (Trp2–5, and His1–5, and -7), were also well represented. Genes involved in arginine biosynthesis (*ARG*1, -7 and -5,6) were the most downregulated (more than five-fold) in this group.

Interactions of negatively regulated genes in transmembrane transport group were represented at medium confidence level (Figure 5B). Most of the highly-interconnected genes were related to mitochondria. Some were connected to respiratory chain and ATP synthesis (e.g., CYT1, RIP1, COX5A, COX15, OLI1, and PET9), transporters of amino acids and their biosynthesis intermediates (MMP1, MUP1, MCH4, VBA4, RTC2, GNP1, BAP2 and -3, AGP1, AQR1, and VBA4), phosphate transporters (PHO84 and PHO90), transporters of divalent metal ions of iron, copper, zinc (FTR1, SMF3, CCC2, and COT1), ABC transporters (AUS1, SNQ2, and PDR12) and other multidrug transporters (QDR3, ERC1, and YCR023C). Thus, majority of genes were involved in transport of amino acid, consistent with downregulated biosynthesis of these molecules.

### 2.5. Link between Host Gene Expression Altered by Viral DsRNA and Cell Sensitivity to K2 Toxin

We compared the list of differently expressed genes of virus hosting cells, with the genes conferring increased resistance or sensitivity of target cell to the K2 killer toxin [23]. There were 42 common genes, dispersed in upregulated and downregulated processes, as well as those involved in an increased toxin susceptibility and resistance. Elimination of the M-2 virus deprives cells of both K2 toxin production and immunity to the toxin. M437 [L+M−] cells boost expression of genes, whose absence confers increased sensitivity for the K2 toxin (*LTV1*, *BUD23*, *NSR1*, *FYV5*, *FYV7*, *LRP1*, *MRT4* and *HGH1*). The majority of those upregulated genes are related to ribosomes and RNA processing. In line with previous observation, part of genes conferring increased resistance of target-cell to K2 toxin in corresponding knockout strains were downregulated upon M-2 dsRNA elimination (namely *BIO3*, *MCR1*, *COR1*, *COX7*, *COX15*, *CWH41*, *VTC2*, *SEL1*, *SNZ1*, *ARR2*, *YCP4* and *ECM1*). These negatively regulated genes are involved in several processes (e.g., oxidation-reduction, membrane trafficking, glycosylation, and ER-associated protein degradation). Genes, whose deletions were shown to confer either increased susceptibility (*VPS41*, *SNX41*, *SIT1*, *ARO1*, *SCS7*, *GCV1*, *CAF17* and *YPR013C*) or resistance (*YOR010C*, *YOL136C*, *YOR154W*, *DIE2*, *FKH1*, *ARG4*, *SML1* and *ISU1*) to K2 toxin, were preferentially downregulated upon elimination of both L-A-lus and M-2 viruses. 

## 3. Discussion

In this study, we addressed the impact of dsRNA viruses on transcriptional status of native strain M437 [L+M+]. Prolonged infection of yeast with L-A-lus and M-2 viruses resulted in versatile coadaptation of viruses and host, thus the strains representing virus-naive conditions—either M-2 free or L-A-lus and M-2 free—were prepared manually. In such a way, transcriptional alterations of wild type strain M437 were described from the perspective of dsRNA-cured cells. We measured transcript levels using RNA-Seq, a robust and an extremely sensitive standard for analysis of global gene expression [23,42,43,44]. We have demonstrated that curing cells from either M-2 or L-A-lus simultaneously with M-2 resulted in moderate alterations of host gene expression. 

**Figure 6:**
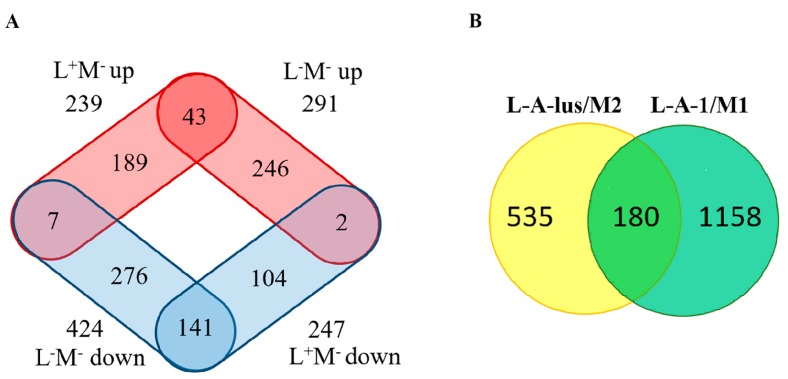
Number of genes differently expressed in virus-free cells: (**A**) Response to the loss of M2 dsRNA (L+M−) or both L-A-lus and M2 dsRNA’s (L−M−). Red color represents set of upregulated genes, blue-downregulated. (**B**) Overlap of genes impacted by L-A-1/M1 (green color) and L-A-lus/M2 viruses (yellow).

The Venn diagram represents numbers of differently expressed genes identified in M437 [L+M−] and M437 [L−M−] cells (Figure 6A).

Elimination of M-2 dsRNA alone led to exclusive upregulation and downregulation of 189 and 104 genes, respectively. Combined removal of the L-A-lus and M-2 dsRNAs resulted in unique upregulation and downregulation of 246 and 276 genes, respectively, suggesting that L-A-lus virus has a more profound impact on host gene expression. M437 [L+M−] cells share number of downregulated and upregulated genes (141 and 43, respectively) with M437 [L−M−] cells. It might be the consequence of co-adaptation and additive effects of small transcriptional changes induced by the elimination of both dsRNA viruses. There was also a small number of differently expressed genes that were oppositely regulated in M437 [L+M−] and M437 [L−M−] cells, further confirming high compatibility of L-A-lus and M-2 dsRNA viruses.

M-2 dsRNA is responsible for toxin and immunity-ensuring component production. Maintenance of K2 toxin demands higher cellular energy production; thus, M-2 removal should be associated with a relief for a host cell by lowering energy costs for synthesis of viral RNA and proteins. Propagation of L-A-lus dsRNA and capsid proteins in host cells also requires more resources than completely cured, dsRNA-free cells. Our study uncovers the processes related to amino acid and lipid biosynthesis, transport and energy production to be positively regulated upon infection with L-A-lus and M-2 dsRNAs, whereas ribosome biogenesis and stress responses are downregulated following the infection. This observation is consistent with the notion that killer cells could be more competitive and less stress-aware due to the presence of the host protection system. It is also possible that dsRNA viruses might have yet unrevealed roles in controlling of cellular RNA metabolism and other processes, not linked directly to the maintenance of a virus in the cell.

Upregulation or downregulation of genes do not necessarily mean that elimination of viral dsRNA is altering “normal” gene expression levels. It might be just the opposite, when the presence of virus is attenuating or boosting the expression of certain genes. In this case, transcription of genes related to ribosome biogenesis and RNA processing could be normally suppressed, while amino acid biosynthesis might be stimulated to produce killer toxin and capsid proteins in natural K2 killer cells. Ribosome biogenesis might be repressed in native K2 killer strain for mobilizing of the host cell resources for killer toxin maintenance while following the M-2 elimination it returns to normal state. Alternatively, elimination of the M-2 dsRNA might change the L-A-lus induced transcription to compensate the loss of M-2, thus upregulating the ribosome biogenesis. From the perspective of L-A-lus virus, M-2 acts like a parasite by utilizing resources from the host and helper virus. It has been demonstrated that dsRNA satellite benefits from the drop in the L-A virus copy number [14,45]. Critical dependence of yeast virus propagation on the concentration of ribosomal content was previously reported, suggesting that most *mak* mutations affect M-1 virus propagation by targeting the supply of proteins from the L-A virus and that the translation of the L-A mRNA depends critically on the amount of free 60S ribosomal subunits [46]. Indeed, in our study, *MAK5*, *MAK11*, *MAK16* and *PET18* genes, required for maintenance of M-1 dsRNA, were found upregulated exclusively in the presence of the L-A-lus dsRNA and absence of M-2 dsRNA, in agreement with altered transcription of these four genes reported previously [36].

The magnitude of the impact of dsRNA viruses on gene expression of *Saccharomyces cerevisiae* observed in our study was consistent with findings of microarray data published by McBride et al. (2013) [36]. The alterations of host gene expression affected by L-A-lus and M2 viruses were moderate, usually not exceeding the limit of four-fold change. Similarly, the loss of L-A-1 and the M-1 viruses resulted in the change to host gene expression pattern, mostly not exceeding three-fold [36]. We found that 180 genes, detected in the analysis of L-A-lus and M2 viruses, significantly affecting gene expression, overlapped with uncovered in the L-A-1/M1 study, while the other 535 genes were uniquely upregulated and downregulated in our study (Figure 6B). Comparative analysis of strains possessing different viral systems found the enriched GO terms of positively regulated genes related to mitochondria functioning, cytosolic and transmembrane transport. However, genes associated with organic acid metabolism, including amino acid metabolism, were affected differently. In response to the loss of L-A-lus and M2 viruses, small molecule metabolic process was downregulated, while, in L-A-1 and the M-1 viruses-free cells, overexpressed. Typical differences were highlighted for biosynthesis of valine, leucine and isoleucine (Figure S3). Such discrepancy could be attributed to different viral systems in different strains targeted, as well as alternative experimental techniques, e.g., RNA-Seq and microarray, employed. 

Long lasting coadaptation of dsRNA viruses and host cells led to moderate transcriptional responses induced by the elimination of viral dsRNA. Disruption of tightly linked killer system by removing either M or L-A along with M dsRNAs resulted in stress response, alterations in the biosynthesis of ribosomes, lipids and amino acids. Our study is the first attempt to employ high-throughput RNA-Seq. for evaluation of the impact of individual mycoviral dsRNAs on baking yeast gene expression. The roles of M and L-A dsRNAs in the individual biological pathways of the cell have been deciphered. Insights on alteration of host gene expression will help to understand the biology of dsRNA mycoviruses and their impact on the host cells.

## 4. Materials and Methods 

### 4.1. Yeast Strains and Culture Media

*S. cerevisiae* strain M437 (*wt HM/HM* [*kil-K2*]) [47], harboring L-A-lus and M-2 dsRNA viruses, and isogenic strains M437 [L+M−] (*wt, HM/HM [kil-0]*) and M437 [L−M−] (*wt, HM/HM [kil-0]*), cured of the M-2 dsRNA and both L-A-lus and M-2 viruses, respectively, were used for gene expression profiling. *S. cerevisiae* yeast strain į’1 (MATį *leu2-2* [*kil-0*]) was used as the sensitive strain for testing of killing phenotype [48].

Yeast cells were grown in standard YPD medium (1% yeast extract, 2% peptone, 2% dextrose, and 2% agar). To test the K2 activity, MBA medium (0.5% yeast extract, 0.5% peptone, and 2% dextrose), adjusted to pH 4 with 75 mM phosphate-citrate buffer and supplemented with 0.002% methylene blue dye, was used.

### 4.2. Curing Yeast Strain from M-2 DsRNA

M-2 dsRNA was eliminated from the M437 cells by means of moderate heat treatment. Yeast cells were spread onto YPD-agar plates and incubated at 37 °C for 4–5 days. The surviving colonies were replica-plated on MB-agar plates overlaid with sensitive to K2 toxin *S. cerevisiae* strain į’1 and grown for 2 days at 25 °C. Colonies lacking killer phenotype (not forming lysis zones) were selected for dsRNA isolation and visual inspection by gel electrophoresis. Absence of M-2 dsRNA was also confirmed by 2-step RT-PCR. 

### 4.3. Curing Yeast Strain from L-A-lus DsRNA

*S. cerevisiae* strain M437 was transformed with pYAK-G-LA-1gag [49] expression vector with truncated version of the L-A-1 *GAG* gene. Constitutive overexpression of the truncated *GAG* resulted in both L-A-lus and M-2 dsRNAs elimination from yeast cells. The absence of viral genome in yeast was examined and confirmed by agarose gel electrophoresis of dsRNA and by RT-PCR. The absence of killing phenotype was confirmed by the killing assay. The L-A-1 *GAG* expression vector was eliminated by several passages for 24 h in liquid YPD medium. Elimination of vector was confirmed by non-growth of yeast cells on selective YPD medium supplemented with 200 μg/mL of G418 (geneticin) and by PCRs with primers specific for pYAK-G-LA-1gag vector (5′-CATTAGAAAGAAAGCATAGC and 5′-TCATGTAAGGACTGCAAG) and for *GAG* gene sequences (5′-CGGAATTATGTCGTCTC and 5′-TCATGTAAGGACTGCAAG). PCR cycling parameters consisted of an initial denaturation at 98 °C for 30 s; following as 30 cycles of 98 °C for 10 s, 58 °C for 20 s and 72 °C for 45 s; and a final extension step at 72 °C for 5 min.

### 4.4. Detection of Killing Phenotype

K2 toxin producing yeast strain M437 [L+M+] and yeast colonies after the dsRNA curing process were spotted onto MBA medium seeded with sensitive yeast strain į’1 (1 × 10^6^ cells/plate). Plates were incubated for 2 days at 25 °C. Non-growth zones around the colonies tested were attributed to the presence of killing phenotype [50].

### 4.5. Total RNA and DsRNA Extraction

Yeast cells were grown in liquid YPD medium at 25 °C for 16 h with shaking at 250 rpm. Collected cells (2 × 10^8^ cells) were washed with 1 mL 50 mM EDTA solution, resuspended in 1 mL 50 mM TrisCl pH 8.8 containing 1% ā-mercaptoethanol and incubated at room temperature (RT) for 15 min. Cells were precipitated and mixed with 800 ģL TES (0.1 M NaCl, 0.01 M Tris-Cl pH 7.5, 0.01 M EDTA, 0.2% SDS) solution, then 600 ģL phenol added and incubated for 30 min by using moderate shaking. The upper aqueous phase, separated by centrifugation at 15,000 *g* for 30 min, was treated with 60 ģL 3M NaAc pH 5.2 and 660 ģL 2-propanol. The pellet was collected by centrifugation at 15,000 *g* for 10 min, washed with cold 75% ethanol and resuspended in 16 ģL of nuclease-free water. For DNA elimination, additional incubation with DNAse I at 37 °C for 30 min was performed. DNAse I was inactivated by adding EDTA to final concentration of 5 mM and incubating at 65 °C for 10 min. Total RNA was used for cDNA synthesis and subsequent PCR reaction for checking M-2 and L-A-lus dsRNA presence. After total RNA isolation, rRNA and other single-stranded RNAs were subsequently removed by precipitation with 2.8 M LiCl for 16 h at 4 °C and centrifugation at 15,000 *g* for 45 min at 4 °C. Double-stranded RNR was precipitated from the aqueous phase by ethanol [51]. 

### 4.6. Detection of L-A-lus and M-2 DsRNAs by 2-step RT-PCR

Total RNA was used as a template for cDNA synthesis carried out with RevertAid First Strand cDNA Synthesis Kit (Thermo Fisher, Vilnius, Lithuania), according to the manufacturer’s instructions. L-A-lus-specific primers (5′-CATGCATTTAAAAAGTTCTGGAC and 5′-GTGCTAACTAGAGCATGTGTAAGG) and M-2-specific primers (5′-GGGAAAAAATGAAAGAGACTACCACCAG and 5′-GGGCTAGCCGCTGTCACATTC) accordingly were used in cDNA synthesis. The same primers were used to detect L-A-lus and M-2 virus specific cDNAs conducting PCRs with DreamTaq DNA Polymerase (Thermo Fisher, Vilnius, Lithuania). PCR cycling parameters for L-A-lus detection consisted of an initial denaturation at 95 °C for 3 min; following as 30 cycles of 95 °C for 30 s, 54 °C for 30 s and 72 °C for 2 min; and a final extension step at 72 °C for 5 min. The following thermocycler conditions were used for M-2 dsRNA detection: 95 °C initial denaturation for 3 min, followed by 30 cycles of 95 °C for 30 s, 72 °C for 80 s; and a final extension step at 72 °C for 5 min. PCR products analyzed by electrophoresis on 1% agarose gel.

### 4.7. Preparation of Total RNA for Next-Generation Sequencing

The total RNA was isolated using GeneJET RNA Purification Kit (Thermo Fisher, Vilnius, Lithuania), according to the Yeast Total RNA Purification Protocol. DNA from total RNA samples was eliminated by DNAse I (Thermo Fisher, Vilnius, Lithuania) treatment at 37 °C for 30 min. RNA quantity and integrity were primarily evaluated by agarose gel electrophoresis (see Figure S1). Additionally, RNA quantity and quality was assessed by Macrogene Inc., Seoul, Korea (https://dna.macrogen.com/) using the Agilent Technologies 2100 Bioanalyzer prior proceeding to RNA-Seq. The RNA integrity number (RIN) of total RNA was greater than 8.0.

### 4.8. RNA Sequencing and Data Analysis

cDNA library construction, quality control and sequencing were performed by Macrogene Inc., South Korea (https://dna.macrogen.com/). The cDNA libraries were constructed and sequenced for three independent biological replicates with 100 bp paired-end reads on the Illumina HiSeq 2000 platform (Macrogen Inc., Seoul, Korea). All resulting RNA-Seq data have been made available in GEO, with accession number GSE100290. 

Prior to assembly, reads were subjected to trimming and filtering using CLC Genomics Workbench v. 8.5 (CLC Inc., Aarhus, Denmark). Low quality reads were discarded (quality limit of 0.01). We also removed: ambiguous nucleotides (ambiguities 1), adapter sequences and sequences less than 50 nucleotides in length. Reads were mapped to the *S. cerevisiae* S288C reference genome (NCBI reference sequence: GCF_000146045.2_R64_genomic_20170309) with the following alignment parameters: maximum number of mismatches 2; minimum length fraction 0.9; minimum identity within the mapped sequence 0.95; maximum number of best-scoring hits for a read 30. The expression values for each transcript were calculated as Reads Per Kilobase of transcript per Million mapped reads (RPKM). The “Exact Test” for two-group comparisons was implemented and the Empirical analysis of DGE tool was applied. Transcripts with expression fold change ≥1.5 and an FDR-corrected *p*-value ≤ 0.05 (false-discovery rate of 5%) were chosen for further analyses.

Specific gene functions and biological pathways were annotated according to SGD (Saccharomyces Genome Database, http://www.yeastgenome.org). Differentially expressed transcripts were subjected to term enrichment analysis using GOTermFinder (http://go.princeton.edu/cgi-bin/GOTermFinder) [52]. Significance *p* values were calculated with the hypergeometric test, using the Benjamini and Hochberg false discovery rate (FDR) correction for the enrichment of each GO term. Fold enrichment (F.E.) was determined by dividing the frequency of specific gene cluster to the total frequency for each GO term.

The protein network was created by STRING v. 10 [53] and imported in Cytoscape v. 3.5.1 [54,55]. Associations between proteins are represented by thick lines based on chosen confidence score (0.5, medium; 0.7, high; or 0.9, highest confidence level). The Venn diagram was created manually, comparing set of genes differently expressed in M437 [L−M−] and M437 [L+M−] cells as well as in A364AXS7 K1 killer strain and virus–cured cells [36]. Amino acids metabolic pathways were drawn using as a reference the Kyoto Encyclopedia of Genes and Genomes (KEGG) database [56].

### 4.9. QRT-PCR Analysis

Quantitative PCRs for *FYV7*, *ARO10* and *TAF10* genes were performed using Luminaris HiGreen qPCR Master Mix (Thermo Fisher, Vilnius, Lithuania), and the protocol according to the manufacturer’s instructions. The following primer pairs were used: *TAF10* (5′-CCTATCATTCCCGATGCAGT-3′ and 5′-AGCTCTCGCCTGACTGTTGT-3′), *ARO10* (5′-CCTGGTGATGTTGTCGTTTG-3′ and 5′-TGAGCGTTTGAGTGGTCTTG-3′), *FYV7* (5′-GGGTACAGCCAAGCAAAATC-3′ and 5′-ATTGCCCTGGCTTCCTTAAT-3′). All measurements were taken in triplicates. The expression ratios were calculated using the 2^−ÄÄCT^ method [57]. 

## Supplementary Materials

The following are available online at www.mdpi.com/2072-6651/9/8/233/s1. Figure S1: Quality control of RNA extraction and elimination of L-A-lus and M-2 viral dsRNA from independently isolated clones, Figure S2: qRT-PCR validation of *FYV7* and *ARO10* expression compared to the RNA-Seq values for [L+M−] and [L+M+] cells, Figure S3. Fragment of amino acid metabolism pathways. Pathways constructed according to KEGG database [56], Table S1: The list of differentially expressed genes in response to the loss of either M-2 dsRNA or both L-A-lus and M-2 dsRNA viruses, Table S2: GO terms in biological process for genes with altered expression level in response to elimination of viral RNAs, Table S3: GO terms in molecular functions for genes induced or repressed in host cells with eliminated viral RNAs, Table S4: GO terms in cellular localization of genes induced or repressed in host cells with eliminated viral RNAs.
