# Supplementary material for: Different Metabolic Pathways Are Involved in Response of Saccharomyces cerevisiae to L-A and M Viruses
Source: Toxins (Basel). 2017 Jul 25;9(8):233. doi: 10.3390/toxins9080233 (PMC5577567; doi:10.3390/toxins9080233)
Supplement: Supplementary file 1 [file toxins-09-00233-s001.zip › toxins-210554-supplementary/supplementary figures.pdf]

Supplementary Materials: Different Metabolic Pathways Are Involved in Response of *Saccharomyces cerevisiae* to L-A and M Viruses

Juliana Lukša, Bazilė Ravoitytė, Aleksandras Konovalovas, Lina Aitmanaitė, Anzhelika Butenko, Vyacheslav Yurchenko, Saulius Serva and Elena Servienė

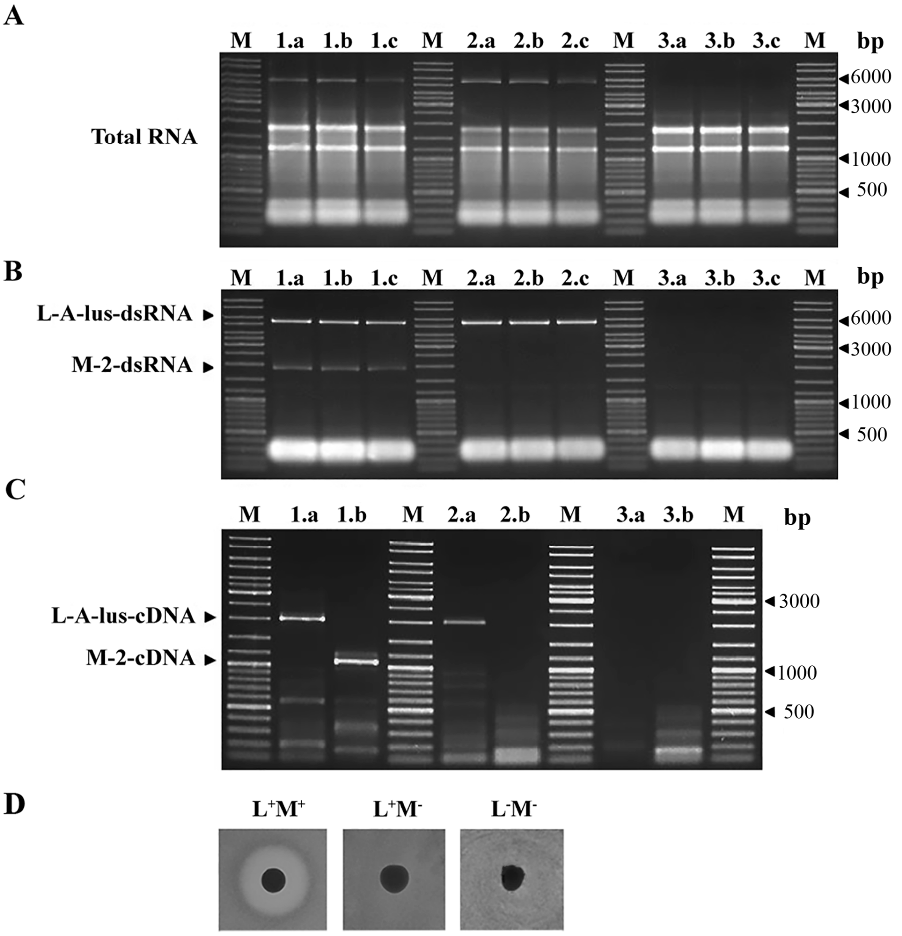

Figure S1. Quality control of RNA extraction and elimination of L-A-lus and M-2 viral dsRNA from independently isolated clones. (**A**) Total RNA preparations for RNA-Seq. analysis. Total RNA extracted from independent clones of three generated cell types: M437 [L+M+] (indicated as 1.a, 1.b, 1.c); M-2-dsRNA-free M437 [L+M−] (indicated as 2.a, 2.b, 2.c) and both dsRNAs-free M437 [L−M−] (marked as 3.a, 3.b, 3.c). (**B**) dsRNA purified from different cell types. Independent clones marked as in A. (C) 2-step RT-PCR product analysis. Independent clones marked as in (**A**). L-A-lus-cDNA and M-2-cDNA correspond to the cDNA products of L and M viruses, respectively. M: molecular weight (kb) marker GeneRuler DNA Ladder Mix (Thermo Fisher, Vilnius, Lithuania). (D) Killing assay: M437 [L+M+] cells showed growth inhibition of sensitive *S. cerevisiae* α'1 cells, M437 [L+M−] and M437 [L−M−] did not demonstrate lysis zones on overlay of α'1 cells.

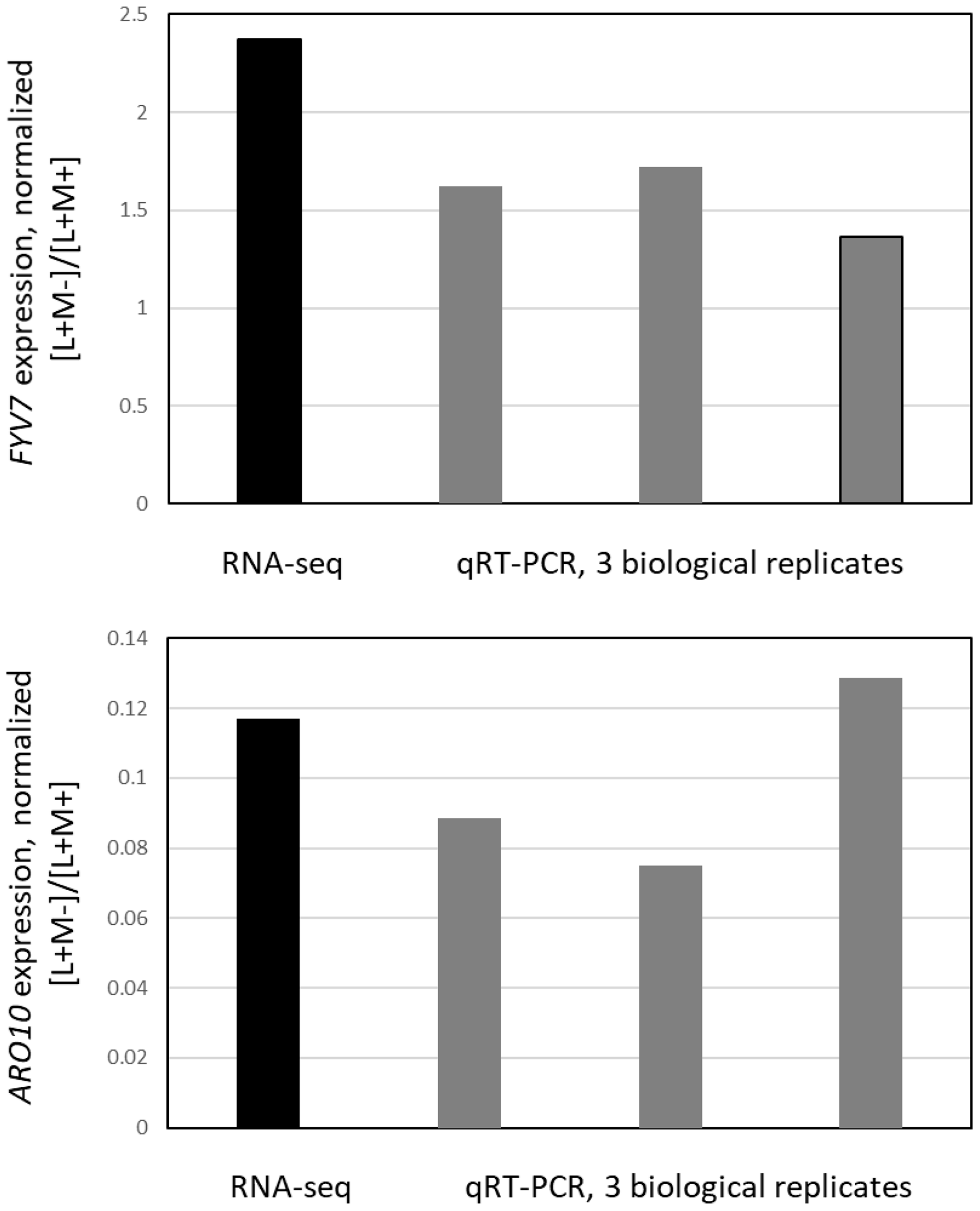

Figure S2. qRT-PCR validation of *FYV7* and *ARO10* expression compared to the RNA-Seq. values for [L+M−] and [L+M+] cells. Data for three independent biological replicates are presented separately. Each bar is an average of three technical replicates. *TAF10* expression was used for normalization.

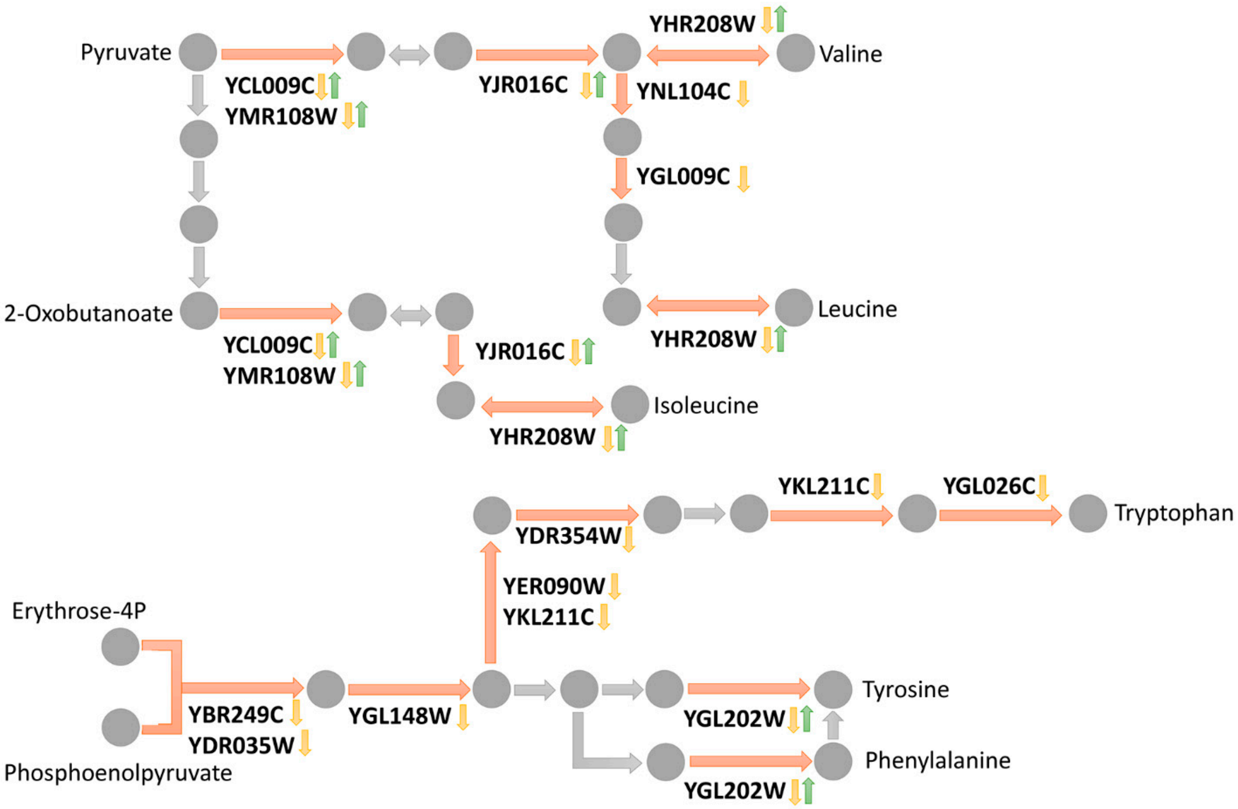

Figure S3. Fragment of amino acid metabolism pathways. Pathways constructed according to KEGG database [1]. Gene names are in bold, primary substrate and product names are next to the gray circles. Arrows next to the gene names depict expression changes in L-A-lus M2 viruses-free cells (yellow arrow) and in L-A-1 M1 viruses-free cells (green arrow) vs virus-infected cells. Direction of the arrow corresponds to the transcript level.

References

1 Kanehisa, M.; Furumichi, M.; Tanabe, M.; Sato, Y.; Morishima, K. KEGG: New perspectives on genomes, pathways, diseases and drugs. *Nucleic Acids Res.* **2017**, *45*, D353–D361, doi:10.1093/nar/gkw1092.
